# Comparative mRNA and LncRNA Analysis of the Molecular Mechanisms Associated With Low Silk Production in *Bombyx mori*

**DOI:** 10.3389/fgene.2020.592128

**Published:** 2021-01-21

**Authors:** Jinghua Ruan, Meiyu Wu, Xiaogang Ye, Shuo Zhao, Jianshe Liang, Lupeng Ye, Zhengying You, Boxiong Zhong

**Affiliations:** ^1^College of Animal Sciences, Zhejiang University, Hangzhou, China; ^2^College of Environmental and Resource Sciences, Zhejiang University, Hangzhou, China; ^3^College of Life Sciences and Medicine, Zhejiang Sci-Tech University, Hangzhou, China

**Keywords:** *Bombyx mori*, silk production, RNA-seq, lncRNA, posterior silk gland

## Abstract

Naked pupa sericin and Naked pupa are two mutant strains of *Bombyx mori* with extremely low or no fibroin production compared to the Qiufeng and Baiyu strains, both of which exhibit very high silk fibroin production. However, the molecular mechanisms by which long non-coding RNAs regulate fibroin synthesis need further study. In this study, we performed high-throughput RNA-seq to investigate lncRNA and mRNA expression profiles in the posterior silk gland of Qiufeng, Baiyu, Nd-s^D^, and Nd silkworms at the third day of the 5th instar. Our efforts yielded 26,767 novel lncRNAs and 6,009 novel mRNAs, the expression levels of silk protein genes and silk gland transcription factors were decreased in Qiufeng vs. Nd-s^D^ and Qiufeng vs. Nd, while those of many genes related to autophagy, apoptosis, RNA degradation, ubiquitin-mediated proteolysis and heat shock proteins were increased. Moreover, the expression of a large number of genes responsible for protein synthesis and secretion was significantly decreased in Nd. GO and KEGG analysis results showed that nucleotide excision repair, mRNA surveillance pathways, amino acid degradation, protein digestion and absorption, ER-associated degradation and proteasome pathways were significantly enriched for the Qiufeng vs. Nd-s^D^ and Qiufeng vs. Nd comparisons. In conclusion, our findings contribute to the lncRNA and mRNA database of *Bombyx mori*, and the identified differentially expressed mRNAs and lncRNAs help to reveal the molecular mechanisms of low silk production in Nd-s^D^ and Nd, providing new insights for improvement of silk yield and elucidation of silk mechanical properties.

## Introduction

*Bombyx mori* of *Lepidoptera* is an ideal and economical model insect for scientific research because of its ease of feeding, high fecundity and short life cycle (Zhu Xs and Xu, 2009). Qiufeng and Baiyu are two economical silkworm strains with large cocoons, high yields and excellent mechanical properties. In contrast, two other strains, the silk yield phenotypic mutants Naked pupa (Nd) (Takei et al., 1984) and Naked pupa sericin (Nd-s^D^) (Inoue et al., 2005), exhibit extremely low or no fiber production.

Non-coding sequences were once considered to be the “junk” sequences of the genome because they do not have protein-coding functions (Balakirev and Ayala, 2003). With in-depth study of the functions and mechanisms of microRNAs (miRNAs), it has gradually become recognized that non-coding RNAs (ncRNAs) are important regulators of protein-coding genes. NcRNAs are now generally named based on their locations in cells and on their sizes. A large number of ncRNA transcripts were identified in a large-scale sequencing study of the mouse transcriptome; that study also suggested the existence of long non-coding RNAs (lncRNAs) (Okazaki et al., 2002). However, further research was not conducted for a long time due to limitations in functional annotation techniques. The development of high-throughput sequencing technology has made large-scale lncRNA research covering the whole genome possible. LncRNA is a type of RNA transcript that is >200 nucleotides in length and does not encode a protein. LncRNAs and mRNAs are both transcribed by RNA polymerase II, and their transcription and cleavage mechanisms are very similar; most of these sequences also contain 5′ cap and 3′ polyA tail structures. However, compared with mRNAs, lncRNAs exhibit relatively low expression, strong tissue specificity, limited homology among sequence families, and poor conservation across species (Derrien et al., 2012).

In recent years, studies have shown that lncRNAs are involved in various biological processes, and the mechanisms of gene expression regulation mediated by lncRNAs can be roughly divided into the following three categories: (1) epigenetic modification, which involves regulation of downstream genes by histone modification (Gong and Maquat, 2011) and chromatin remodeling (Beniaminov et al., 2008; Zhao et al., 2008; Mercer et al., 2009); (2) transcriptional regulation, which involves effects on the transcription of protein-coding genes (upregulation or downregulation of the expression of the genes) via interactions with their promoters or transcription factors (Igor et al., 2007; Zhao et al., 2008; Jing et al., 2010); and (3) posttranscriptional regulation, which involves processing of lncRNAs into small RNAs such as siRNAs (Tiash and Chowdhury, 2018; Tsai et al., 2018) and miRNAs (Yuya et al., 2008; Annilo et al., 2009; Pandey et al., 2018) that function via complementary binding to coding gene transcripts. Recent research on lncRNAs has mainly concentrated on humans and mice. Little research has been performed on insect ncRNAs, especially lncRNAs; furthermore, research focused on small ncRNAs, such as research on the functional annotations of miRNAs and siRNAs, and on the prediction of their target genes is still not very extensive. However, studies have shown that lncRNAs can facilitate multiple biological processes in *Drosophila*, such as sex determination (Mulvey et al., 2014), male courtship behavior (Chen et al., 2011) and X-chromosome gene regulation (Ilik et al., 2017; Samata and Akhtar, 2018).

Thus far, research on the silk gland development process has mostly concentrated on protein-coding genes. Similar to studies on other species, most *Drosophila* ncRNA investigations have explored the regulation of gene expression by miRNAs and have focused less on the regulation of gene expression by lncRNAs. In previous studies, Li et al. (2011, 2014) identified 189 novel intermediate-size ncRNAs in the silkworm and speculated that some of the ncRNAs were to be involved in the regulation of silk gland development. Two of nine silk gland-enriched ncRNAs, Bm-102 and Bm-159, might play roles through epigenetic modification. A total of 11,810 lncRNAs were identified using RNA-seq, and coexpression network analysis revealed that hub lncRNAs associated with the middle silk gland (MSG) and posterior silk gland (PSG) may function as regulators of the biosynthesis, translocation, and secretion of silk proteins (Wu et al., 2016). The differentially expressed lncRNAs (DELs) in the silk gland are mainly associated with processes related to silk protein translation, which indicates that lncRNAs may participate in the regulation of silk protein production between domestic and wild silkworms (Zhou et al., 2018). Furthermore, lncRNAs play an important role in silk gland-related apoptosis by interacting with other RNAs (Chen et al., 2019).

In the current study, we performed high-throughput Illumina sequencing to systematically identify lncRNAs and differentially expressed genes (DEGs) in the PSG tissues of silkworms with high and extremely low silk fibroin production. Our efforts yielded a large number of silk gland lncRNAs and related target genes and revealed that some of the lncRNAs may serve as precursors of miRNAs. In this study, 26,767 novel lncRNAs and 6,009 novel mRNAs were identified, which identified more lncRNAs than previous RNA-seq studies. and greatly expanded the silkworm lncRNA database. Among them, 4,606 of identified lncRNA transcripts are Nd or Nd-s^D^ strain-specific, which can be used as candidates to study the mechanism of low silk production. We further explored the potential regulatory functions of lncRNAs on the development of PSGs and the biosynthesis of fibroin. Our current study indicated that the function of the silkworm lncRNAs is not only related to fibroin synthesis and apoptosis of silk glands as stated in the previous studies (Wu et al., 2016; Zhou et al., 2018; Chen et al., 2019), but also promote the degradation of silk proteins by regulating RNA/amino acid degradation, the proteasome and proteolysis. Moreover, lncRNA may participate in nucleotide excision repair, heat shock proteins expression, autophagy activation and endoplasmic reticulum stress response to maintain the homeostasis of the PSG cells. Our study described more comprehensively than ever the elements that may affect the development of PSG and silk protein synthesis, as well as the potential regulatory function of lncRNA on these factors. These results enable a better understanding of the molecular mechanisms of PSG development and fibroin synthesis and thus provide a theoretical basis for improvement of the mechanical properties of silk.

## Results

### Morphology of the PSG and Cocoon

**Figure 1:**
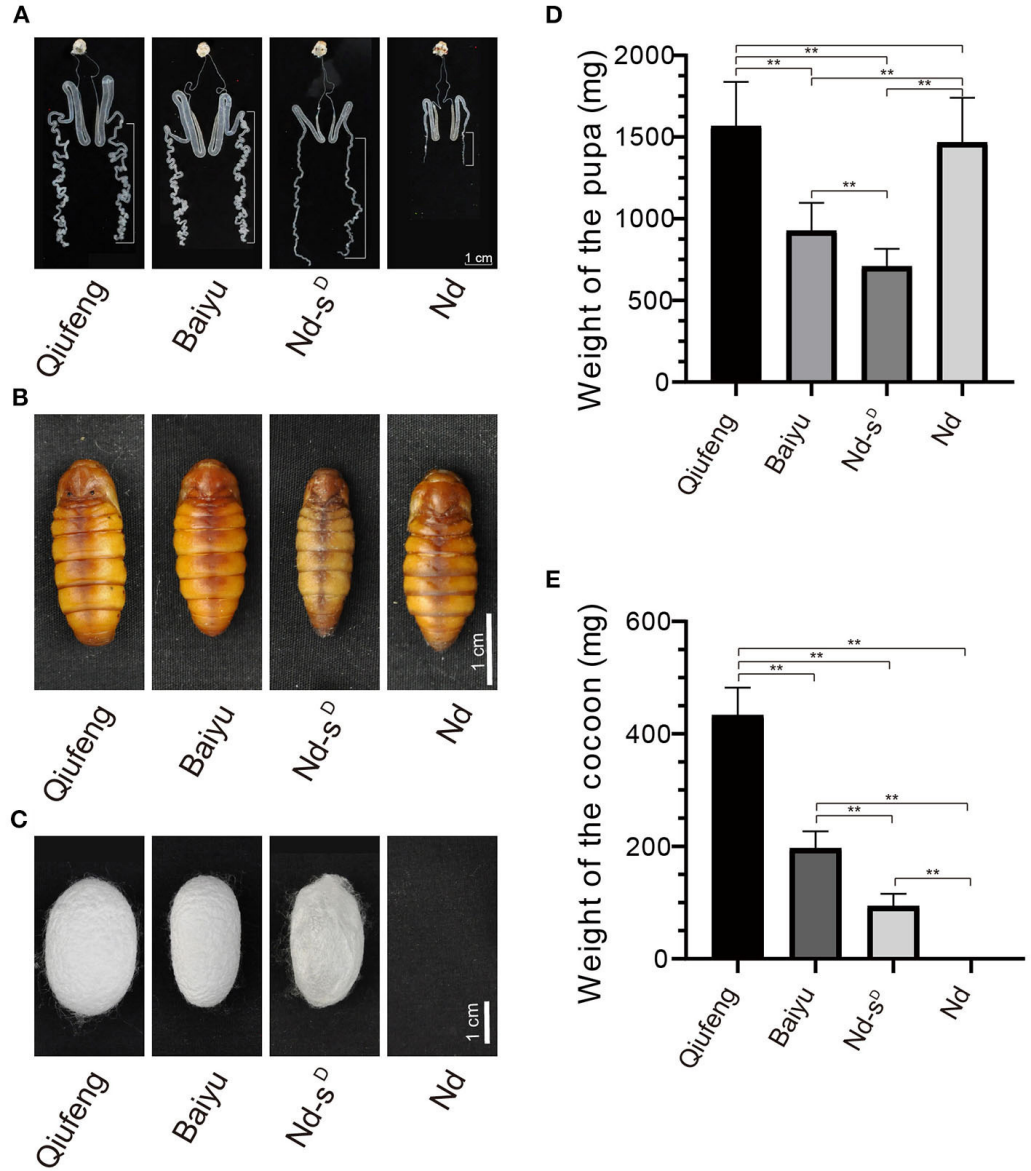
Comparison of Qiufeng, Baiyu, Nd-s^D^ and Nd. The dissected silk glands of silkworms at I5D3. The white long scale bars mark the PSG **(A)**. The pupa of Qiufeng, Baiyu, Nd-s^D^ and Nd from left to right **(B)**. The cocoon of Qiufeng, Baiyu, Nd-s^D^ and Nd from left to right **(C)**. Comparison of the weight of the pupa **(D)** and cocoon shell **(E)** of Qiufeng, Baiyu, Nd-s^D^ and Nd. Sixteen pupae and cocoons were collected for each species including eight males and eight females, and Student's *t*-test was performed to analyze their pupa and cocoon shell weight differentials. **P < 0.01. Scale bars, 1 cm in **(A–C)**.

The silk gland is a vital organ for the production of silk fibroin and is divided into three segments according to morphology and function, namely, the anterior silk gland (ASG), the MSG and the PSG. As shown in Figure 1, the PSGs of Nd and Nd-s^D^ silkworms at the third day of the 5th instar (I5D3) were severely degraded and atrophied, while those of Qiufeng and Baiyu silkworms were long and folded (Figure 1A). The morphology of the pupa was significantly different in different strains (Figure 1B), the weight of pupa of Qiufeng strain was the heaviest, followed by Baiyu and Nd-s^D^. However, the pupa weight of Nd was close to that of Qiufeng strain due to the dysfunction of silk protein secretion (Figure 1D). Moreover, Nd-s^D^ spun only very thin and easily torn cocoons mainly composed of sericin, called sericin cocoons, and Nd failed to spin normal cocoons such that they eventually formed naked pupae (Figure 1C). The weight of cocoons of different strains was significantly different, among which Qiufeng strain had the heaviest cocoon, followed by Baiyu and Nd-s^D^ (Figure 1E).

### Transcriptome Assembly and LncRNA Identification

**Figure 2:**
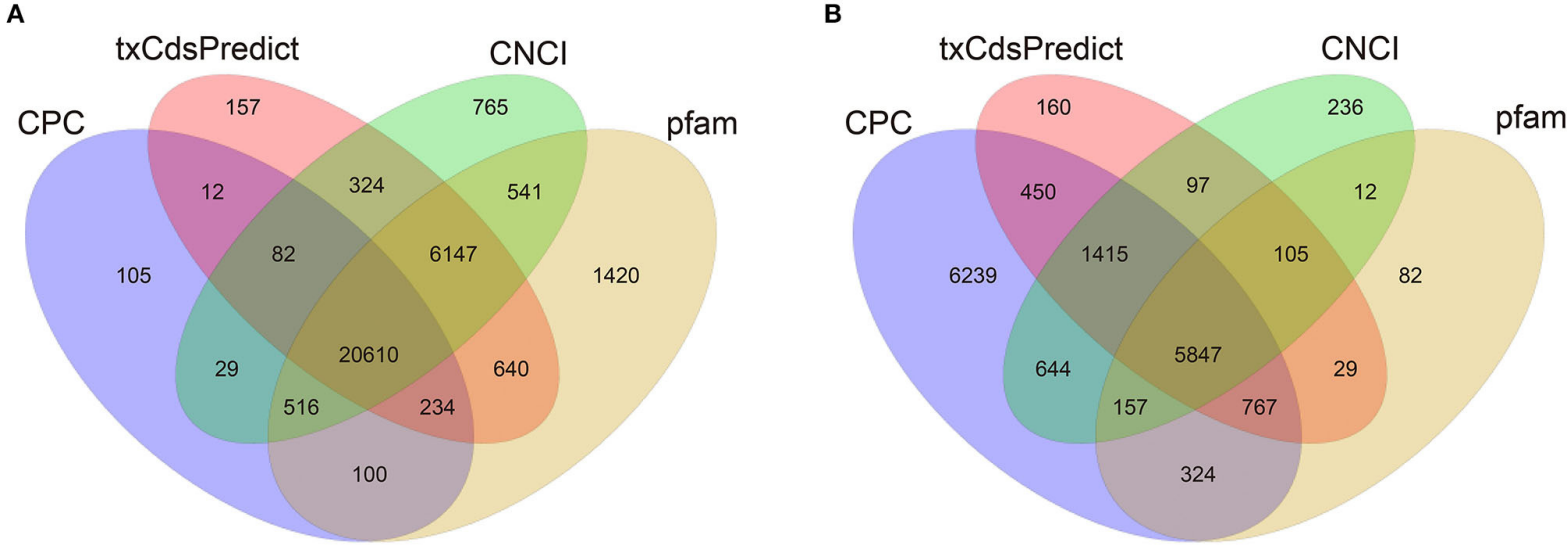
Predicted lncRNAs and mRNAs of the PSG in *Bombyx mori* as filtered with CPC, txCdsPredict, and CNCI and compared with the Pfam database. Venn diagram of the lncRNAs **(A)**. Venn diagram of the mRNAs **(B)**.

To investigate the molecular mechanisms of mRNAs and lncRNAs in PSG development and fibroin synthesis, four cDNA libraries were constructed, and 96,750,098, 96,750,306, 96,749,478 and 96,748,404 clean reads were obtained in the Qiufeng, Baiyu, Nd-s^D^ and Nd libraries, respectively. The clean data were mapped to the reference genome (*Bombyx mori*) using HISAT, and 76.81, 76.57, 78.96, and 79.70% of the sequences were successfully mapped for the Qiufeng, Baiyu, Nd-s^D^ and Nd libraries, respectively. These data indicated that the four libraries were of good quality, that the numbers of reads obtained were large, and that most of the reads could be matched to the genome. The raw sequence reads was upload to Sequence Read Archive (SRA) (http://www.ncbi.nlm.nih.gov/) with accession PRJNA634502. In this study, 16,069 SilkDB-annotated mRNAs were all mapped from the assembled transcripts, and a total of 37,531 novel transcripts were identified (Supplementary Table 1). Since a true protein-coding transcript is more likely than a non-coding transcript to harbor a long, high-quality open reading frame (ORF), the coding potential of novel transcripts was predicted with the software programs CPC, txCdsPredict, and CNCI and the Pfam protein database. Herein, 27,589 and 8,291 transcripts were predicted to be isoforms of 26,908 novel lncRNA genes, and 6,009 novel mRNA genes, respectively (Figure 2). Among them, 141 unique transcripts can be compared to the existing lncRNA collected in Wu et al. (2016) and BmncRNAdb (Supplementary Table 2). Finally, 26,767 novel lncRNA genes were obtained. It is worth noting that 4,606 lncRNAs were specifically expressed only in Nd or Nd-s^D^ (Supplementary Table 3).

### Genomic Characteristics of the LncRNAs

In order to further study the genomic characteristics of the lncRNAs expressed in PSG tissues, we conducted a comparative analysis of the exon numbers, transcript numbers, and transcript lengths of all lncRNAs and mRNAs identified in this study (Supplementary Figure 1). Unlike the mRNAs, most lncRNAs were single-exon lncRNAs, while a smaller proportion were two-exon lncRNAs. LncRNAs with lengths between 0-500 nt and 500-1000 nt accounted for ~90% of the total transcripts with lengths shorter than those of mRNAs; thus, the lengths of the lncRNAs were mainly within 1000 nt. Most lncRNAs encoded only one transcript, while some lncRNAs encoded multiple transcripts. In contrast, many mRNAs encoded multiple transcripts. In this study, the lncRNAs had fewer exons, fewer transcripts, and shorter transcript lengths than the mRNAs, similar to the results obtained for lncRNAs in other species. In addition, the lncRNAs exhibited lower expression levels than the mRNAs (Supplementary Figure 1).

### Differential Expression Analysis

**Figure 3:**
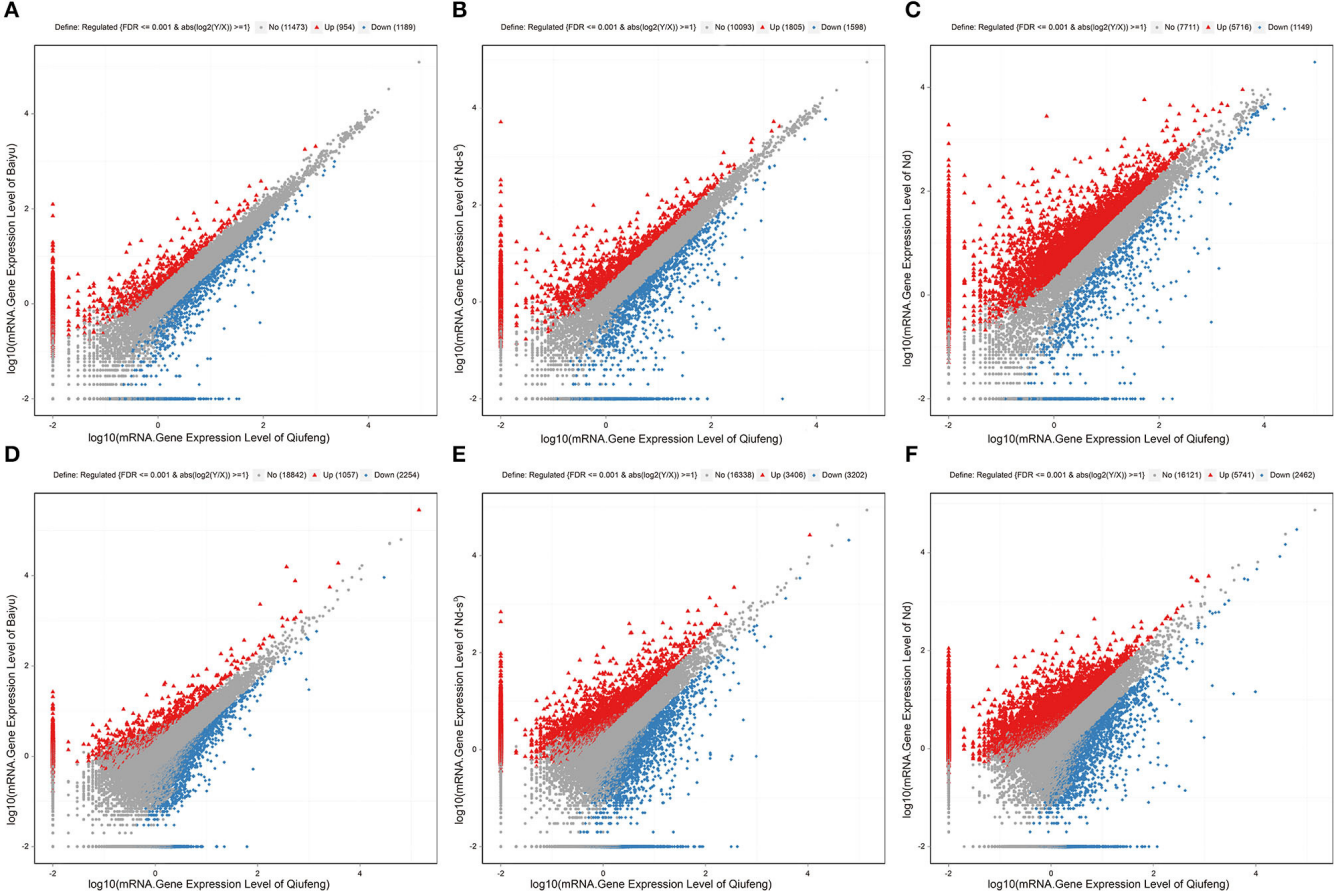
Scatter plot of mRNA and lncRNA expression levels. mRNA gene expression levels in Qiufeng vs. Baiyu **(A)**, Qiufeng vs. Nd-s^D^ **(B)** and Qiufeng vs. Nd **(C)**. LncRNA gene expression levels in Qiufeng vs. Baiyu **(D)**, Qiufeng vs. Nd-s^D^ **(E)** and Qiufeng vs. Nd **(F)**. The red points represent upregulated DELs, the blue points represent downregulated DELs, and the gray points represent genes that were not significantly different between strains.

**Figure 4:**
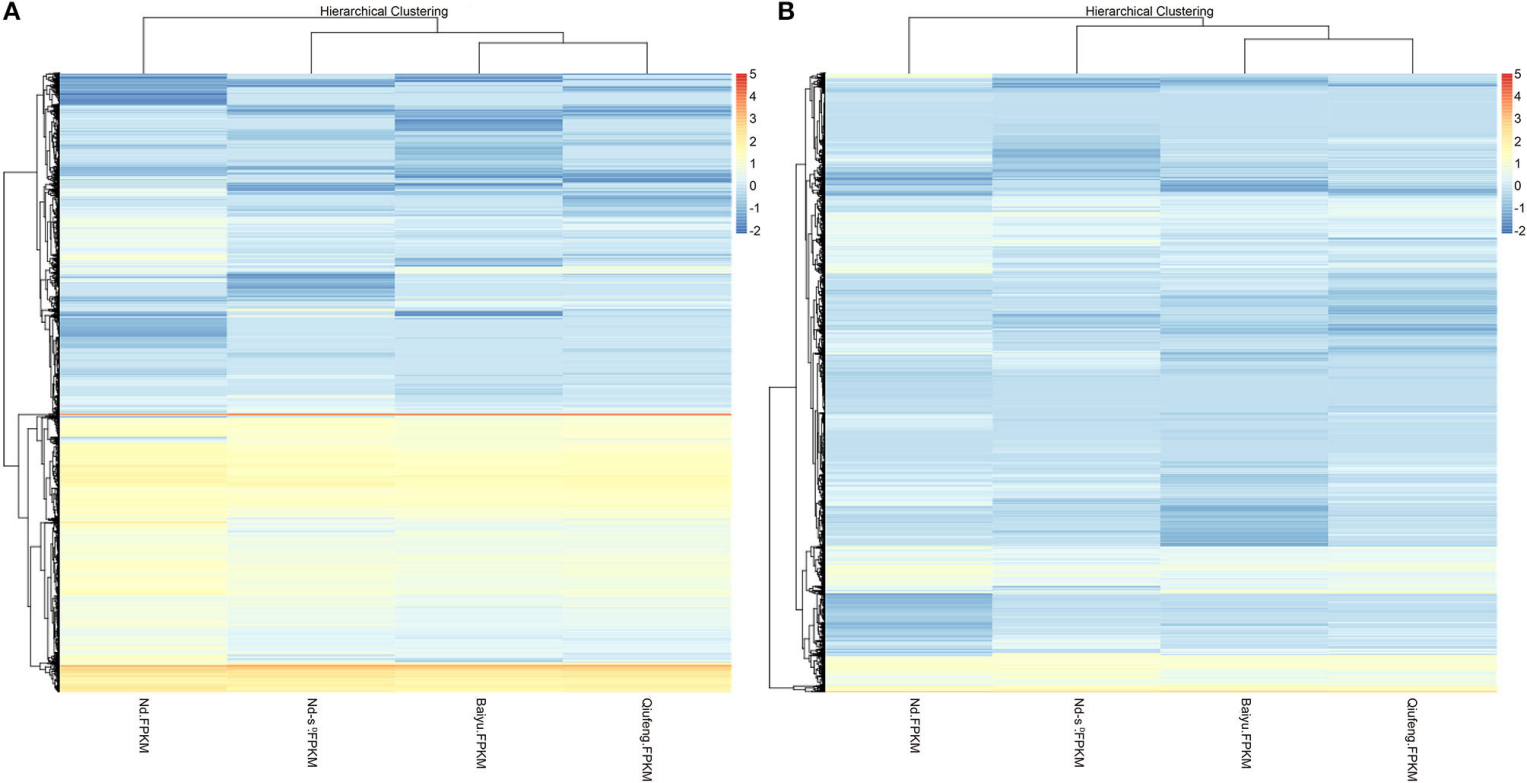
Hierarchical clustering of mRNAs **(A)** and lncRNAs **(B)** in Qiufeng, Baiyu, Nd-s^D^ and Nd.

There were 2,143 DEGs (Supplementary Table 4) and 3,311 DELs (Supplementary Table 5) in the Baiyu library compared with the Qiufeng library. Among these genes and lncRNAs, 954 genes and 1,057 lncRNAs were upregulated, and 1,189 genes and 2,254 lncRNAs were downregulated. A total of 3,403 genes and 6,608 lncRNAs were differentially expressed in the Nd-s^D^ library relative to the Qiufeng library (Supplementary Tables 6, 7). Among these, 1,805 genes and 3,406 lncRNAs were upregulated, including 637 Nd-s^D^ strain-specific genes, and 1,598 genes and 3,202 lncRNAs were downregulated. In addition, 6,865 and 8,203 DEGs and DELs were identified between the Qiufeng library and the Nd library, respectively (Supplementary Tables 8, 9). In the Nd library compared with the Qiufeng library, 5,716 and 5,741 genes and lncRNAs were significantly upregulated, respectively, including 1,055 Nd strain-specific genes, 1,149 and 2,462 genes and lncRNAs were significantly downregulated. Scatter plots were drawn to represent the DEGs and DELs (Figure 3). Furthermore, to determine the primary patterns of gene expression, hierarchical clustering analysis of all DEGs and DELs was further conducted based on the FPKM values using pheatmap (Figure 4).

We further compared the DEGs in Qiufeng vs. Baiyu, Qiufeng vs. Nd-s^D^, and Qiufeng vs. Nd. The expression of fibroin genes (*FibH, FibL, p25*) in the Nd strains was significantly downregulated, and the fold change in *FibH* was >6-fold. The expression of *SGF2* and *Bmdimm* was significantly decreased in Nd-s^D^, and the expression of *Bmdimm* was also lower in Qiufeng vs. Nd than in Qiufeng vs. Baiyu, and 23 rRNA genes related to protein biosynthesis were downregulated (Table 1). Many genes related to RNA degradation, ubiquitin-mediated proteolysis, Hsps, autophagy, and apoptosis were upregulated in Nd. Three upregulated genes related to RNA degradation were identified in the PSG in Nd-s^D^, and many upregulated DEGs were also identified as being related to ubiquitin-mediated proteolysis, autophagy and apoptosis (Supplementary Table 10).

**Table 1:** Differential expression genes associated with the biosynthesis of silk protein.

**Category**	**GeneID**	**Description**	**log2(Baiyu/Qiufeng)**	**log2(Nd-s^*D*^/Qiufeng)**	**log2(Nd/Qiufeng)**
Fibroin genes and silk gland factor	BMSK0016068	FibH	0.3	0.2	−6.4
	BMSK0008215	FibL	0.4	0.0	−1.6
	BMSK0001060	P25	0.5	0.0	−2.7
	BMSK0000690	SGF2	0.3	−2.2	−0.1
	BMSK0010050	Bmdimm	−1.7	−12.5	−3.8
Ribosomal protein	BMSK0008917	Mitochondrial 28S ribosomal protein S14	0.5	0.7	−11.5
	BMSK0001399	Ribosomal protein L37A	−0.4	−0.1	−1.6
	BMSK0012680	Ribosomal protein L37	−0.2	−0.5	−1.6
	BMSK0007500	39S ribosomal protein L27, mitochondrial	−0.6	1.1	−1.5
	BMSK0013084	Ribosomal protein L26	−0.5	0.0	−1.4
	BMSK0001949	Ribosomal protein L35	−0.2	−0.1	−1.4
	BMSK0005571	Ribosomal protein S15A	−0.6	−0.2	−1.4
	BMSK0001352	Ribosomal protein L11	−0.5	0.1	−1.3
	BMSK0013426	60S ribosomal protein L38	−0.3	0.1	−1.3
	BMSK0009998	40S ribosomal protein SA	−0.1	0.0	−1.3
	BMSK0001139	Ribosomal protein L34	−0.4	−0.7	−1.3
	BMSK0010922	Ribosomal protein S9	−0.3	−0.1	−1.3
	BMSK0008533	Ribosomal protein L5	−0.3	−0.3	−1.2
	BMSK0012298	Ribosomal protein L36	−0.6	0.0	−1.2
	BMSK0014093	Ribosomal protein L32	−0.3	0.4	−1.1
	BMSK0003283	Ribosomal protein L15	−0.2	0.0	−1.1
	BMSK0006276	40S ribosomal protein S28	−0.6	0.1	−1.1
	BMSK0013364	Ribosomal protein S30	−0.4	−0.2	−1.1
	BMSK0006809	Ribosomal protein L36A	−0.1	0.1	−1.1
	BMSK0007331	Mitochondrial ribosomal protein S21	−0.9	−0.8	−1.1
	BMSK0008678	Ribosomal protein L8	−0.3	−0.1	−1.0
	BMSK0001031	40S ribosomal protein S21	−0.5	0.0	−1.0
	BMSK0000895	60S ribosomal protein L23	−0.2	−0.2	−1.0

*FibH, silk fibroin heavy chain gene; FibL, silk fibroin light chain gene; P25, fibroin P25; SGF2, silk gland factor 2*.

### Analysis of Pre-miRNAs

LncRNAs may function as small RNA precursors; the long transcripts can be sequentially cleaved by Drosha and Dicer into multiple smaller RNAs, and each mature miRNA can perform its own function in specific subcellular structures (Wilusz et al., 2009). Mature miRNAs can act on multiple sites, inhibiting translation processes and affecting gene silencing, so identifying miRNAs and their target genes can aid in understanding of the PSG regulatory process. A total of 1,292 predicted positions on 961 lncRNAs were compared to miRNA precursors and were found to correspond to 183 miRNAs (Supplementary Table 11). Previous studies have confirmed that bmo-miR-275 has negative effects on the expression of *SGF1* and *BmSer-2*, and *SGF1* is an important transcription factor regulating the expression of *FibH, Ser-1* and *P25*. Another study on miRNAs in the PSG has predicted that *FibH* can be targeted by bmo-mir-3334 and that bmo-miR-305 can simultaneously target *FibL* and *SGF1*. Notably, 11 lncRNAs were found to have the potential to be processed into mature bmo-mir-2774, which can target *SGF3*.

### LncRNA Functions in Cis-/ Trans-Regulation

The potential target genes of lncRNAs acting in cis/trans were predicted in order to investigate lncRNA functions. A total of 17,874 lncRNAs were screened as potential regulators of 18,048 mRNAs within 10 kb upstream and 20 kb downstream, forming a total of 12,931 lncRNA-mRNA pairs (Supplementary Table 12). We sought to further elucidate the cis-regulatory mechanisms and identified 3,516 pairs of lncRNAs and mRNAs that partially or fully overlapped with each other. The forms of overlap could be divided into ten types. Interaction analysis of complementary lncRNAs-mRNAs was conducted to explore the trans actions of lncRNAs using RNAplex and produced 2,177 lncRNA-mRNA interaction pairs (Supplementary Table 12). The regulation of lncRNAs and mRNAs is not one-to-one, as lncRNAs can regulate one or more mRNAs at the same time; similarly, mRNAs can be regulated by multiple lncRNAs. Further investigation indicated that 811 lncRNAs were located on chromosome 25. Of particular interest was the observation that the fibroin gene *Fib H*, which has been confirmed to be responsible for the naked pupa phenotype in a recent study (Hu et al., 2019b) resided within the 10 kb upstream/20 kb downstream of seven lncRNAs. LncRNAs were also screened in the 10 kb upstream and 20 kb downstream of *FibL* and *p25*, intimating that synthesis of silk fibroin may be regulated by lncRNAs and adjacent protein-coding genes. In addition, silk gland transcription factors involved in transcriptional regulation of fibroin genes were detected, and three lncRNAs were identified in the 20 kb downstream of *SGF1* and *SGF2* (Table 2).

**Table 2:** Target gene related with fiber protein of lncRNA in cis regulation.

**Description**	**GeneID**	**LncRNA_class**	**LncRNA**	**log2(Baiyu/Qiufeng)**	**log2(Nd-s^**D**^/Qiufeng)**	**log2(Nd/Qiufeng)**
FibH	BMSK0016068	cis_mRNA_up10k	LTCONS_00035325	0.1	0.7	−4.8
		cis_mRNA_dw20k	LTCONS_00036259	0.4	−0.2	−5.7
		cis_mRNA_dw20k	LTCONS_00036260	0.6	1.0	−5.3
		cis_mRNA_dw20k	LTCONS_00036262	1.9	0.3	−5.5
		cis_mRNA_dw20k	LTCONS_00036263	0.3	0.4	−8.4
		cis_mRNA_dw20k	LTCONS_00036264	1.7	−0.5	−2.6
		cis_mRNA_dw20k	LTCONS_00036266	0.7	−0.4	−4.6
FibL	BMSK0008215	cis_mRNA_dw20k	LTCONS_00013360	0.1	−7.3	−7.3
P25	BMSK0001060	cis_mRNA_dw20k	LTCONS_00024660	0.2	−0.2	−1.9
		cis_mRNA_dw20k	LTCONS_00024661	−0.3	−0.8	−1.2
		cis_mRNA_up10k	LTCONS_00024663	6.2	6.1	0.0
SGF1	BMSK0014952	cis_mRNA_dw20k	LTCONS_00036348	−1.4	−0.1	−3.1
SGF2	BMSK0000690	cis_mRNA_dw20k	LTCONS_00002622	−0.2	−1.5	−1.1

*FibH, silk fibroin heavy chain gene; FibL, silk fibroin light chain gene; P25, fibroin P25; SGF1, silk gland factor 1; SGF2, silk gland factor 2*.

We further studied the target genes of DELs in Qiufeng vs. Baiyu, Qiufeng vs. Nd-s^D^ and Qiufeng vs. Nd (Table 3). We detected a large number of upregulated target genes involved in autophagy and apoptosis in both Nd-s^D^ and Nd. Apart from this, two target genes related to RNA degradation and eight target genes related to ubiquitin-mediated proteolysis were differentially upregulated in Nd-s^D^; more upregulated target genes were also identified in RNA degradation and ubiquitin-mediated proteolysis, and 14 differentially downregulated rRNA genes related to protein biosynthesis were identified in Nd.

**Table 3:** Expression levels of the target genes involved in synthesis and degradation of protein.

**Category**	**GeneID**	**Description**	**log2(Baiyu /Qiufeng)**	**log2(Nd-s^*D*^/Qiufeng)**	**log2(Nd /Qiufeng)**
Autophagy	BMSK0000510	Autophagy-related protein 9A	0.0	5.2	6.6
	BMSK0000705	piggyBac transposable element-derived protein 3-like	−1.1	3.7	1.7
	BMSK0005327	Tetratricopeptide repeat protein 27	−0.8	−0.2	1.1
	BMSK0005367	Extracellular regulated MAP kinase	1.6	3.3	2.9
	BMSK0006994	Phosphatidylinositol 3-kinase 60	0.0	0.5	2.3
	BMSK0007445	TRAF6	−0.3	−0.5	1.2
	BMSK0008608	Rab7	−0.4	−0.4	1.0
	BMSK0008882	Ras-related GTP-binding protein A [Papilio polytes]	−1.4	0.7	2.5
	BMSK0010120	Cathepsin B precursor	−0.1	−0.3	2.2
	BMSK0013395	cAMP-dependent protein kinase C1	−0.2	−0.2	1.5
	BMSK0014018	Transposase IS4	−4.2	−4.2	2.6
	BMSK0014659	Autophagy-related protein 2 homolog A	0.8	−0.2	1.2
	BMSK0001223	Serine/threonine-protein phosphatase 2A catalytic subunit beta	0.2	0.3	1.9
	BMSK0001868	RB1-inducible coiled-coil protein 1 isoform X2	6.2	8.3	8.9
	BMSK0004105	Ras-like protein 2	0.4	0.1	2.9
	BMSK0004511	Autophagy related protein ATG1C	0.0	6.0	4.5
Apoptosis	BMSK0006994	Phosphatidylinositol 3-kinase 60	0.0	0.5	2.3
	BMSK0007251	Dihydropyridine-sensitive l-type calcium channel [Operophtera brumata]	−0.4	−0.2	2.1
	BMSK0010120	Cathepsin B precursor	−0.1	−0.3	2.2
	BMSK0009900	Actin 3a	1.8	3.1	3.7
	BMSK0009901	Actin 5c	0.0	15.0	16.3
	BMSK0015743	Actin, partial [*Zygaena filipendulae*]	−2.2	−1.0	2.6
	BMSK0015085	biorientation of chromosomes in cell division protein 1-like 1	0.8	0.9	2.2
	BMSK0015756	Growth arrest and D damage-inducible protein GADD45 alpha	−0.2	2.1	4.3
	BMSK0001343	Cytochrome c	−0.7	0.9	1.4
	BMSK0004001	Eukaryotic translation initiation factor 2-alpha kinase-like	6.2	8.7	10.2
	BMSK0005073	Nuclear factor NF-kappa-B p105 subunit	−5.5	2.4	2.4
	BMSK0005367	Extracellular regulated MAP kinase	1.6	3.3	2.9
	BMSK0007319	Cactus, partial	0.1	1.4	1.4
RNA degradation	BMSK0005676	Protein Tob1	4.5	5.4	7.7
	BMSK0011209	TM2 domain-containing protein almondex	−0.7	−0.2	1.2
	BMSK0011731	Putative nuclease HARBI1 isoform X3	0.5	0.2	1.8
	BMSK0013588	Putative leucine-rich repeat-containing protein DDB G0290503 isoform X2	0.1	0.4	1.7
	BMSK0014887	UPF0692 protein CG33108 isoform X1	0.4	0.0	1.4
	BMSK0015856	Exosome complex component RRP41 [*Papilio xuthus*]	0.2	0.2	1.3
	BMSK0003807	Cell differentiation protein RCD1 homolog	−0.2	0.4	1.3
	BMSK0001678	Enhancer of mR -decapping protein 4	−6.1	2.7	0.7
Ribosomal protein related to synthesis and secretion	BMSK0006276	40S ribosomal protein S28	−0.6	0.1	−1.1
	BMSK0007500	39S ribosomal protein L27, mitochondrial	−0.6	1.1	−1.5
	BMSK0008533	Ribosomal protein L5	−0.3	−0.3	−1.2
	BMSK0008678	Ribosomal protein L8	−0.3	−0.1	−1.0
	BMSK0010922	Ribosomal protein S9	−0.3	−0.1	−1.3
	BMSK0001031	40S ribosomal protein S21	−0.5	0.0	−1.0
	BMSK0012298	Ribosomal protein L36	−0.6	0.0	−1.2
	BMSK0012680	Ribosomal protein L37	−0.2	−0.5	−1.6
	BMSK0013084	Ribosomal protein L26 isoform X1	−0.5	0.0	−1.4
	BMSK0013364	Ribosomal protein S30	−0.4	−0.2	−1.1
	BMSK0014093	Ribosomal protein L32	−0.3	0.4	−1.1
	BMSK0001949	Ribosomal protein L35	−0.2	−0.1	−1.4
	BMSK0006809	Ribosomal protein L36A	−0.1	0.1	−1.1
	BMSK0003283	Ribosomal protein L15	−0.2	0.0	−1.1
Proteasome	BMSK0007235	Protein D7-like [*Amyelois transitella*]	−0.1	0.5	2.3
	BMSK0009832	Proteasome 26S non-ATPase subunit 4	−0.6	0.2	1.4
	BMSK0010504	26S proteasome non-ATPase regulatory subunit 1 [*Amyelois transitella*]	−1.0	0.6	1.4
	BMSK0010503	26S proteasome non-ATPase regulatory subunit 2 [*Amyelois transitella*]	−0.9	0.8	1.6
	BMSK0002154	Proteasome subunit beta 7	−0.6	0.8	2.0
Ubiquitin mediated proteolysis	BMSK0013757	Cullin-2	0.0	6.4	4.0
	BMSK0000620	F-box/WD repeat-containing protein 7 isoform X1	0.0	6.3	5.7
	BMSK0005327	Tetratricopeptide repeat protein 27	−0.8	−0.2	1.1
	BMSK0005746	Ubiquitin-conjugating enzyme E2-24 kDa isoform X1	0.1	0.1	1.2
	BMSK0005862	pre-mR -processing factor 19	−0.8	0.4	1.4
	BMSK0005832	E3 ubiquitin-protein ligase SIAH1-like	−0.5	0.2	1.1
	BMSK0007445	TRAF6	−0.3	−0.5	1.2
	BMSK0007643	Anaphase promoting complex subunit 10	−0.1	−0.2	1.3
	BMSK0011542	NEDD4-like E3 ubiquitin-protein ligase WWP1	4.0	4.8	5.8
	BMSK0010161	E3 ubiquitin-protein ligase SMURF2	−5.5	1.1	2.6
	BMSK0010452	Anaphase-promoting complex subunit 1	2.0	5.6	5.9
	BMSK0010947	SUMO-1 activating enzyme	−0.9	0.5	2.8
	BMSK0011324	RING finger and CHY zinc finger domain-containing protein 1	0.0	1.3	1.2
	BMSK0003443	E3 ubiquitin-protein ligase SIAH1	−0.5	1.2	3.1
	BMSK0003545	F-box and WD-40 domain protein 8	−0.5	−0.3	1.2
	BMSK0003830	Ubiquitin-conjugating enzyme E2-17 kDa	−0.1	0.2	1.4
	BMSK0004700	Ubiquitin-like modifier-activating enzyme 1	0.0	0.6	1.3
	BMSK0010639	Ubiquitin-protein ligase E3 B	−5.6	3.2	−5.6

### GO and KEGG Pathway Enrichment Analysis of DEGs

The DEGs in Qiufeng vs. Baiyu were enriched for 42 GO terms, including the metabolic process, cell, binding and catalytic activity terms. More upregulated DEGs were mapped to the cellular process, metabolic process, binding and biological regulation terms in Qiufeng vs. Nd-s^D^ than in Qiufeng vs. Baiyu. A large number of upregulated DEGs in Qiufeng vs. Nd were enriched not only for the cellular process, metabolic process, binding and catalytic activity terms but also for the biological regulation and response to stimulus terms.

**Figure 5:**
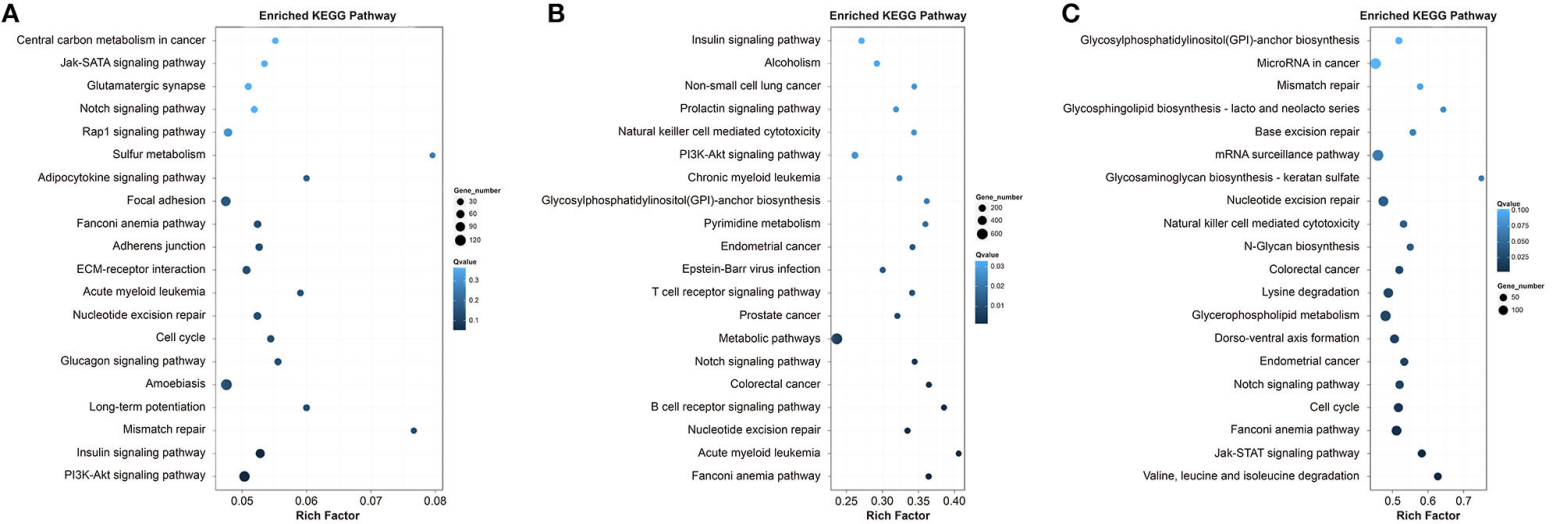
Top 20 KEGG pathways of the DEGs in Qiufeng vs. Baiyu **(A)**, Qiufeng vs. Nd-s^D^ **(B)** and Qiufeng vs. Nd **(C)**. “Rich factor” indicates the ratio of the number of DEGs enriched in the pathway to the total number of genes annotated in the pathway.

KEGG pathway analysis of the DEGs in Qiufeng vs. Baiyu, Qiufeng vs. Nd-s^D^ and Qiufeng vs. Nd were performed, and the top 20 pathways for the three groups are shown in Figure 5. The enrichment results for Qiufeng vs. Baiyu showed that the DEGs were mapped mainly to the focal adhesion pathway and some signaling pathways, such as the PI3K-Akt signaling pathway, the Rap1 signaling pathway, and the Jak-STAT signaling pathway. A majority of DEGs in Qiufeng vs. Nd-s^D^ were enriched in metabolic pathways, pyrimidine metabolism, nucleotide excision repair (NER) and some signaling pathways involved in the regulation of growth and tissue differentiation. In addition, DEGs were significantly enriched in disease-/cancer-related pathways and immune-related pathways, such as the T/B cell receptor signaling pathway and natural killer (NK) cell-mediated cytotoxicity. Among the top 20 pathways in Qiufeng vs. Nd were the cell cycle, notch signaling, cancer-related and natural killer cell-mediated cytotoxicity pathways, in which many mRNAs were enriched. Numerous DEGs were also matched to NER, base excision repair (BER), mismatch repair (MMR) and mRNA surveillance pathways. DEGs in Nd-s^D^ and Nd were significantly enriched in the proteasome pathway and in pathways related to amino acid (valine, leucine, isoleucine, and lysine) degradation. A large number of DEGs in Nd were also enriched in the protein digestion and absorption (*n* = 118) and ubiquitin-mediated proteolysis (*n* = 96) pathways. Notably, protein processing in the endoplasmic reticulum (ER) was a pathway of significant enrichment for both Nd-s^D^ and Nd (Supplementary Table 13).

### GO and KEGG Pathway Enrichment Analysis of the DELs

The target genes of the DELs in the PSG in Qiufeng vs. Baiyu, Qiufeng vs. Nd-s^D^, and Qiufeng vs. Nd were annotated (Supplementary Figure 3). Many downregulated target genes in Qiufeng vs. Baiyu were enriched for the cellular process, metabolic process, cell, macromolecular complex, binding and catalytic activity terms. The upregulated target genes in Qiufeng vs. Nd-s^D^ were mainly annotated with the response to stimulus, development process, growth, and biological adhesion terms. A considerable number of upregulated target genes in Qiufeng vs. Nd were enriched for the cellular process, metabolic process, response to stimulus, biological regulation, binding and catalytic activity terms.

**Figure 6:**
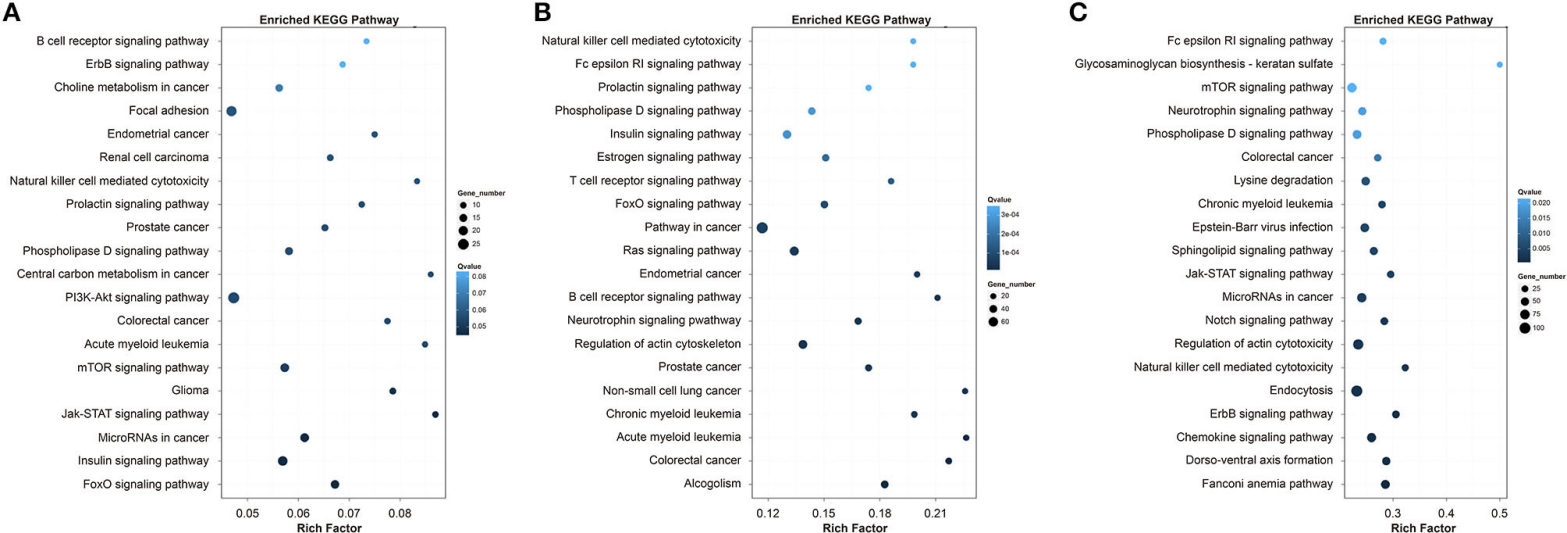
Top 20 KEGG pathways for the target genes of the DELs in Qiufeng vs. Baiyu **(A)**, Qiufeng vs. Nd-s^D^ **(B)** and Qiufeng vs. Nd **(C)**. “Rich factor” indicates the ratio of the number of DEGs enriched in the pathway to the number of genes annotated in the pathway.

The top 20 enriched KEGG pathways for the target genes in Qiufeng vs. Baiyu, Qiufeng vs. Nd-s^D^ and Qiufeng vs. Nd were shown in Figure 6. The target genes in Qiufeng vs. Baiyu were mainly matched to the focal adhesion pathway and to eight signaling pathways. The pathways in which the target genes in Qiufeng vs. Nd-s^D^ were enriched were roughly divided into three categories: disease, signaling and immunity pathways. Among them, eight disease-related pathways were associated mainly with cancer and hematological diseases, and ten signaling pathways were associated with immune responses, including the T/B cell receptor signaling pathway and the Fc epsilon RI signaling pathway. Notably, another enriched pathway, the estrogen signaling pathway, plays an important role in immune regulation in a variety of cancers and vascular diseases. In addition, the target genes were highly enriched in the NK cell-mediated cytotoxicity and actin cytoskeleton regulation pathways. The target genes in Qiufeng vs. Nd were associated with five diseases. Most of the target genes were enriched in seven signal transduction pathways and in the NK cell-mediated cytotoxicity pathway, which mediates inflammation and the immune response and induces programmed cell death. Finally, we found that a large number of target genes were associated with endocytosis (adjusted *p* = 0.00003); NER (adjusted *p* = 0.0015); regulation of autophagy (adjusted *p* = 0.003); the mRNA surveillance pathway (adjusted *p* = 0.006); lysine degradation (adjusted *p* = 0.0003); and valine, leucine and isoleucine degradation (adjusted *p* = 0.034) (Supplementary Table 14). These results were similar to the enrichment results for the DEGs in Qiufeng vs. Nd.

## Discussion

### Expression Profiling of LncRNAs and mRNAs

In this study, RNA-seq was performed to construct RNA libraries, the obtained clean reads were compared to the silkworm genome (SilkDB 3.0) with SOAP, and the transcripts were assembled. In view of the low conservation of lncRNAs across species and tissues, we used three prediction software programs and the Pfam protein database together to predict the coding abilities of the new transcripts in order to reliably distinguish between lncRNAs and mRNAs and to reveal the potential functions of these molecules during PSG development and silk protein synthesis. In this study, 27,589 lncRNAs were compared with previously identified silkworm lncRNAs, and 23,770 novel lncRNAs were identified from the PSG tissues of four silkworm strains. Among them, 4,606 lncRNAs are only expressed in Nd or Nd-s^D^. Compared with protein-coding genes, these lncRNAs had significant differences in exon and transcript numbers and in transcript lengths, and the expression levels of the lncRNAs in all four strains were lower than those of the mRNAs. Wang et al. (2017) and Miao et al. (2018) identified lncRNAs in pigs and goats and similarly found that the lncRNAs exhibited fewer exons and transcripts and shorter transcripts than mRNAs. LncRNAs exhibit similar characteristics across different species, indicating that these characteristics may be important for the biological effects of lncRNAs. In this study, the cluster analysis showed that the mRNAs and lncRNAs expressed in Qiufeng, Baiyu, Nd-s^D^ and Nd were clearly clustered and were classified reasonably well into four clusters. We found that among the mRNAs and lncRNAs, those for Qiufeng and Baiyu clustered closest together; the classification thus indicated that the two strains have high similarity. In contrast, the mRNAs and lncRNAs for Nd formed a separate cluster, indicating that the expression patterns of Nd mRNAs and lncRNAs are significantly different from those of the other three strains. The results of cluster analysis not only intuitively reflected the similarities and differences in PSG mRNAs and lncRNAs among the four silkworm strains but also reflected the objective and reasonable nature of the sequencing data.

We further annotated lncRNAs that were assigned to “unknown regions” in the former analysis. If they are located upstream or downstream of a gene, lncRNAs can interact with cis-acting elements and participate in transcriptional regulation. LncRNAs located in the 3'UTR or downstream of a gene may play other regulatory roles (Carninci et al., 2005). Given that upstream lncRNAs may overlap with promoters or other cis-acting elements of a coexpressed gene, thereby regulating gene expression at the transcriptional or post-transcriptional level (Yazgan and Krebs, 2007; Mercer et al., 2009), we carefully classified the overlapping lncRNAs and target genes to help further elucidate the lncRNA-mediated cis-regulation.

### Analysis of DEGs and DELs

Studies have shown that the silk mechanical properties and cocoon layers of Qiufeng are better than those of Baiyu, so we hypothesized that there would be DEGs in high-yield silkworm strains compared with lower-yield strains. We identified some specific DEGs with high expression levels in Qiufeng, such as the eukaryotic transcription initiation factors eIF2alpha (BMSK0011652) and eIF2beta (BMSK0003276), glycotransferase (BMSK0005817), and leucine tRNA ligase (BMSK0007808), which play important roles in transcription, protein translation and cell proliferation. In addition, *Bmdimm* (BMSK0010050) was also significantly upregulated in Qiufeng. These DEGs may enhance the ability of Qiufeng to synthesize silk proteins through transcription- and metabolism-related pathways.

There were fewer DEGs and DELs in the Qiufeng vs. Baiyu comparison than in the other two comparisons. Interestingly, many of the identified mRNAs and lncRNAs were expressed in only Nd or Nd-s^D^, which suggests that mickle mRNAs and lncRNAs are species-specific. These RNAs could regulate the development of the silk gland through specific spatiotemporal expression and in turn affect the yield and quality of silk.

The DEG analysis showed that the expression of silk protein-encoding genes and transcription factors was significantly downregulated in Nd, suggesting that the formation of naked pupae may be related to a significant decrease in the overall silk protein synthesis capacity. Studies have shown that *SGF2* is an important transcription factor involved in the regulation of *FibL* expression (Ohno et al., 2013; Kimoto et al., 2014), and the expression levels of *SGF2* and *Bmdimm* were significantly reduced in Nd-s^D^ in the current study, indicating that the FibL synthesis ability of Nd-s^D^ is weakened. The sericin cocoon formation of Nd-s^D^ may be related to the decreased transcription factor activity. In addition, lncRNAs located within a range of 10 kb upstream/20 kb downstream of silk protein-encoding genes and silk gland transcription factors were all downregulated in Nd. The expression of LTCONS_00013360, which has a potential cis-regulatory effect on *FibL*, was also significantly downregulated in Nd-s^D^. These associations suggested that these lncRNAs may downregulate the biosynthesis of silk fibroin.

Our analyses revealed that many DEGs and target genes of DELs related to ubiquitin-mediated proteolysis were upregulated in both Nd-s^D^ and Nd. Nd-s^D^ and Nd synthesize a large number of abnormal proteins due to mutations in *FibL* and *FibH*. Ubiquitin-mediated proteolysis is a necessary process for targeted degradation of most short-lived proteins in eukaryotic cells, including cell cycle regulatory proteins and proteins that are not properly folded in the ER, and timely degradation of cell cycle regulatory proteins is important for regulating cell division (Amm et al., 2014). We identified many upregulated DEGs and target genes of DELs that were related to RNA degradation, the proteasome and proteolysis. In addition, the expression of ribosomal proteins involved in protein synthesis and secretion was significantly decreased. These findings indicate that lncRNAs not only exert potential negative regulatory effects on fibroin synthesis but also promote protein degradation. mRNA molecules and ribosomal proteins fulfill indispensable functions in the protein synthesis process; for example, the ribosomal protein S9 is required in the early steps of ribosome biogenesis and is important for silk protein synthesis (Li et al., 2017). mRNA degradation, proteolysis and downregulation of the expression of large amounts of ribosomal proteins will greatly reduce silk production.

Apart from the above results, we found that multiple Hsp genes related to the stress response were upregulated in Nd-s^D^ and Nd, indicating that the PSG cells of Nd-s^D^ and Nd are under stress and that Hsp is essential for homeostasis of the PSG cell environment, protein synthesis, transport, and folding. Unexpectedly, many DEGs and target genes of DELs related to autophagy and apoptosis were also identified in Nd-s^D^ and Nd. However, PSG cell autophagy and apoptosis generally occur in the metamorphic stage; with the discharge of silk fibroin, the PSG gradually shrinks during the spinning process and eventually degrades. Premature activation of the two programmed cell death mechanisms (autophagy and apoptosis) likely causes the PSG tissue to dissociate and disappear earlier than normal, directly affecting the synthesis and secretion of silk protein.

### GO and KEGG Pathway Enrichment Analysis

We performed GO and KEGG pathway enrichment analysis of the DEGs and DELs. DEGs enriched for basic cell processes such as cell metabolism, catalysis, and biological regulation were mainly downregulated in Baiyu compared with Qiufeng, indicating that Baiyu's physiological activity is weakened. In contrast, a large number of genes enriched for these biological processes were upregulated in Nd-s^D^ and Nd, indicating that Nd-s^D^ and Nd have enhanced metabolic activity but that this enhanced metabolic activity is not used to increase silk protein production. Similar enhancement of metabolism in the low-yield strain ZB is used not for the synthesis of silk protein but rather for growth and death (Wang et al., 2016).

KEGG pathway analysis of the DEGs in Qiufeng vs. Baiyu revealed that major genes were enriched in some signaling pathways and that other genes were involved in the regulation of basic cellular processes. The DEGs in Qiufeng vs. Nd-s^D^ and in Qiufeng vs. Nd were mainly enriched in three metabolic pathways associated with cell growth; proliferation; and survival, diseases and immunity. These findings suggest that the mutant strains likely use metabolic modulation to maintain homeostasis and survive. The atrophy and degeneration of the PSG observed in Nd-s^D^ and Nd actually result from malignant transformation. Some previous studies have confirmed that accumulation of damaged proteins caused by mutation/misfolding/aggregation disrupts cell homeostasis and endangers survival under severe stress conditions (Buchberger et al., 2010; Michalak, 2010). Since Nd and Nd-s^D^ are silk fibroin-deficient mutants, many more target genes were mapped to immune/stress-related pathways than to other pathways; differential regulation of such genes enabled the mutants to cope with their silk fibroin deficiency. NK cells participate in early defenses against various forms of stress, such as malignant transformation, among autologous cells. NK cells release cytotoxic granules onto the surfaces of bound target cells, and the effector proteins contained in these granules penetrate the cell membrane and induce programmed death of PSG cells. More DEGs were associated with NER and MMR in Qiufeng vs. Nd-s^D^ and in Qiufeng vs. Nd than in Qiufeng vs. Baiyu. However, BER enrichment was observed only for DEGs in Qiufeng vs. Nd, which implies that severe damage to PSG cells occurs in Nd. These enzymatic pathways are the main cellular mechanisms by which various structural abnormalities (DNA damage) are identified and correct and by which damage is eliminated (Sancar and Reardon, 2004; Li, 2008; Coey and Drohat, 2017). In addition, the mRNA surveillance pathway is a QC mechanism for detection and degradation of abnormal mRNA molecules that includes non-sense-mediated mRNA decay (NMD), non-stop mRNA decay (NSD) and no-go decay (NGD) processes. Among these processes, NMD and NGD were activated in Nd. Target genes were only significantly enriched in the NER and mRNA surveillance pathways in Qiufeng vs. Nd-s^D^ and Qiufeng vs. Nd. It has been demonstrated that the Nd and Nd-s^D^ phenotypes are caused by deletion of exons of *FibH* and *FibL*, respectively. Failure of elimination of damaged DNA or abnormal mRNA molecules can lead to mutagenesis, cancer and cell death, and lncRNAs can participate in activating NER and the mRNA surveillance pathway to eliminate intracellular DNA and mRNA damage. Therefore, we speculate that the PSG cell damage in Nd-s^D^ and Nd directly hinders the synthesis and secretion of silk proteins. The results suggest that lncRNAs expressed in Nd exhibit certain functions that downregulate silk protein synthesis.

It is worth noting that target genes in Nd were also enriched for processes related to degradation of lysine, valine, leucine and isoleucine. These amino acids are indispensable constituents of silk fibroin. We analyzed the amino acid sequence of FibH and found that the four amino acids valine, leucine, isoleucine and lysine accounted for 24.5% (37 aa/151 aa) of the N-terminal domain of FibH. Silkworms synthesize large amounts of silk proteins on the third day of the fifth instar, but a large number of DEGs were enriched in amino acid degradation, protein digestion/absorption, and proteasome pathways in the mutants, suggesting that the synthesized proteins were continuously degraded.

**Figure 7:**
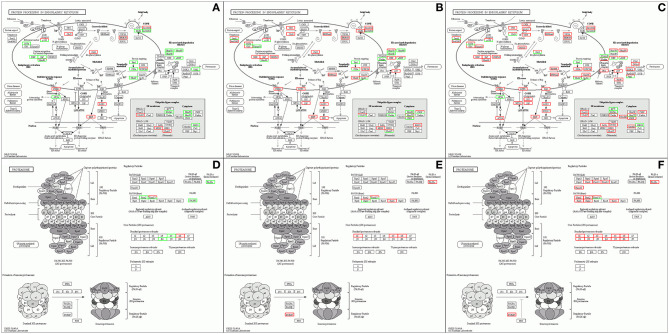
Pathway enrichment analysis of the differentially expressed mRNAs The enrichment results related to protein processing in the ER in Qiufeng vs. Baiyu **(A)**, Qiufeng vs. Nd-s^D^ **(B)** and Qiufeng vs. Nd **(C)** and the enrichment results related to the proteasome in Qiufeng vs. Baiyu **(D)**, Qiufeng vs. Nd-s^D^ **(E)** and Qiufeng vs. Nd **(F)** are shown.

Accumulation of unfolded or misfolded proteins in the ER can cause ER stress. Eukaryotic cells have many different mechanisms to deal with accumulation of unfolded proteins in the ER, such as the unfolded protein response (UPR) (Kudo et al., 2002). Nd-s^D^ alleviates ER pressure by activating the proteins activating transcription factor 6 (ATF6) and JUN N-terminal kinase (JNK) in the UPR reaction to initiate apoptosis. On the one hand, Nd copes with ER stress through the actions of ATF6 and inositol-requiring protein 1 (IRE1). On the other hand, misfolded proteins activate the ER-associated degradation (ERAD) system, which ubiquitinates abnormal proteins that are transferred from the ER to the cytoplasm; the ubiquitinated proteins are then degraded by the proteasome. Similarly, activation of ERAD in Nd-s^D^ helps the mutants to deal with ER stress (Figures 7A–C). We further studied DEGs in the proteasome pathway and found that the activity of regulatory particles and core particles (20S proteasome) was significantly enhanced, indicating that proteasome pathways are activated in Nd-s^D^ and Nd (Figures 7D–F). Additionally, the upregulated target genes of the DELs in Nd were also enriched in the ERAD and proteasome pathways (Supplementary Figure 4), implying that lncRNAs participate in misfolded protein degradation through ERAD and the 26s proteasome to release the pressure on the ER caused by the continuous aggregation of synthesized proteins. ER stress is a prominent feature of many neurodegenerative diseases related to protein misfolding and aggregation, including amyotrophic lateral sclerosis (ALS), Parkinson's disease and Alzheimer's disease (Matus et al., 2011). Other previous studies have revealed consistent results indicating that DEGs in *Bombyx mori* with low silk yield are enriched in neurodegenerative disease pathways (Wang et al., 2014; Hu et al., 2019a). We hypothesize that the ER is under long-term stress due to the continuous accumulation of misfolded FibH, which causes irreversible damage to PSG cells. Nd PSGs gradually atrophy and degenerate, which ultimately leads to naked pupa formation and loss of the ability to synthesize and secrete silk proteins.

## Conclusion

The potential lncRNA- and mRNA-mediated regulation underlying the low silk production in Nd-s^D^ and Nd may be mainly related to four molecular mechanisms. First, decreases in the expression of silk protein genes and their transcription factors may cause the generation of sericin cocoons in Nd-s^D^. These genes are more downregulated in Nd than in Nd-s^D^, and their downregulation eventually leads to naked pupa. Second, the expression of autophagy and apoptosis-related genes is upregulated in Nd-s^D^ and is more significantly upregulated in Nd. mRNAs and lncRNAs activate autophagy and apoptosis in the PSG through cis-regulation and promote dissociation and death of PSG cells. Third, decreases in the levels of genes encoding ribosomal proteins impair protein synthesis in Nd, and the numerous upregulated DEGs and target genes associated with RNA/protein degradation in Nd-s^D^ and Nd indicate that proteins are continuously digested and degraded. Fourth, the truncated FibL and FibH produced by Nd-s^D^ and Nd activate the UPS and ERAD to transfer abnormal proteins from the ER to the cytoplasm, where they are ubiquitinated. These proteins are subsequently degraded by the proteasome. The ER pressure caused by the constant accumulation of mutant FibL and FibH greatly hinders the ability of Nd-s^D^ and Nd PSG cells to synthesize and secrete silk proteins.

## Materials and Methods

### Silkworm Strains and Samples

Domesticated silkworm strains with various quality and yield characteristics were selected in this study to enhance construction of lncRNA libraries and to eliminate bias due to strain specificity. The mutant strains Nd and Nd-s^D^ (Mori et al., 1995; Inoue et al., 2005) and the wild-type strains Qiufeng and Baiyu were reared on fresh mulberry leaves under standard conditions until the third day of the fifth instar. For better lncRNA profiling and eliminating strain-specific effects, the tested samples of Qiufeng and Baiyu each contain 10 PSG tissues from different silkworms, Nd and ND-s^D^ contain 40 PSGs from different silkworms. Each sample contained many silkworm individuals, which helps to minimize the biological error as much as possible. The PSGs were then dissected from the silkworms, washed with precooled 0.7% NaCl and stored in liquid nitrogen until further analysis.

### RNA Isolation, LncRNA Library Preparation, and Illumina Sequencing

The PSGs of all individuals of each strains were mixed together separately, and then ground into powder as a pool for RNA extraction. Total RNA was extracted using TRIzol reagent (Invitrogen, Carlsbad, CA, USA) according to the manufacturer's instructions, with an average concentration of 3391.7 ng/μl. The RNA concentration and quality were measured using a Nano Drop 2000 (Thermo Scientific, Wilmington, DE, USA). Agarose (1%) gel electrophoresis was performed to confirm the integrity of the extracted total RNA, which was then stored at −80°C for future use. All 4 samples had an RNA integrity number (RIN) value >9.0 (Supplementary Table 15).

For lncRNA-seq, a total amount of 3 μg of RNA per sample was used as input material for RNA library construction. After extracting the total RNA, the mRNAs and ncRNAs were enriched by removing rRNA from the total RNA with a kit. The mRNAs and ncRNAs were fragmented into short segments (approximately 200~500 nt) with fragmentation buffer, and first-strand cDNA was then synthesized with random hexamer primers using the fragments as templates. dTTP was replaced with dUTP during synthesis of the second strand. The short fragments were purified and resolved with EB buffer for end reparation and single adenine (A) nucleotide addition. After that, the short fragments were connected with adapters, and the second strands were degraded using uracil-N-glycosylase (UNG) (Parkhomchuk et al., 2009). After agarose gel electrophoresis, suitable fragments were selected for PCR amplification as templates. An Agilent 2100 Bioanalyzer and an ABI StepOnePlus Real-Time PCR System were used for quantification and qualification of the sample libraries in the quality control (QC) steps. Finally, the libraries were sequenced using an Illumina HiSeq^TM^ 2000.

### Quality Analysis, Mapping, and Transcriptome Assembly

We first compared the reads to the ribosomal database using the short reads alignment tool Short Oligonucleotide Analysis Package (SOAP) (Li et al., 2008), allowing up to 5 mismatches. We removed the reads aligned with ribosomal sequences and used the retained data for subsequent analysis. Clean reads were obtained by removing reads with adapters, reads containing poly-N sequences (*N* > 10%), and low-quality reads whose numbers of bases with mass values (Q-values) ≤ 10 accounted for more than 50% of the total reads. The filtered data were aligned to the silkworm genome (https://silkdb.bioinfotoolkits.net) using the software HISAT2 (v2.0.4, parameters: –phred64 –sensitive –no-discordant –no-mixed -I 1 -X 1000) (Kim et al., 2015). Then, the reads were assembled with StringTie (v1.0.4, parameters: -f 0.3 -j 3 -c 5 -g 100 -s 10000 -p 8) (Pertea et al., 2015). All the reconstructed transcripts were compared with known mRNA and lncRNA sequences to obtain information about their positional relationships. Regarding the assembly results for a single transcript, we required each transcript to meet the following requirements: (1) a fragments per kilobase of exon per million mapped fragments (FPKM) value ≥0.5, (2) a *coverage* value >1, and 3) a length>200. Classification and statistical analysis of the lncRNAs were conducted with the NONCODE database (Bu et al., 2011). Finally, we used Cufflinks gffread software (v2.2.1, parameter: -p 12) to generate the transcript sequence assembled by StringTie (Pertea et al., 2015), and the merged transcripts were retained for subsequent analysis.

### Sequence Analysis and LncRNA Identification

After transcript reconstitution, the coding abilities of the transcripts were predicted to distinguish between mRNAs and lncRNAs using four software programs, the Coding Potential Calculator (CPC) (Lei et al., 2007), txCdsPredict, and the Coding-Non-Coding Index (CNCI) (Liang et al., 2013), and the Pfam database (Finn et al., 2015). All three prediction software programs scored the coding abilities of the transcripts and then set a scoring threshold to distinguish between lncRNAs and mRNAs. Transcripts that could be compared to the Pfam protein database were considered to be mRNAs, while the others were defined as lncRNAs. Only when the results of at least three of the four judgment methods were consistent did we consider that a transcript was definitively an mRNA or a lncRNA. The scoring thresholds for the three software programs were as follows: CPC, 0 (transcripts with a value >0 were considered mRNAs, and those with a value <0 were considered lncRNAs); CNCI, 0 (transcripts with a value >0 were considered mRNAs, and those with a value <0 were considered lncRNAs); and txCdsPredict, 500 (transcripts with a value >500 were considered mRNAs, while those with a value <500 were considered lncRNAs). All predicted lncRNAs were aligned to lncRNAs collected in BmncRNAdb and previous study (Wu et al., 2016; Zhou et al., 2018). We introduced NCBI Blast tool for the comparison which was based on sequence similarity (Parameters: blastall -e 1e-5 -p blastn -a 8 -d xx.fa -i xx.fa -o blast). Only those blast hits with %identity >90% and % query_coverage >85% were considered to be already annotated transcripts.

### Analysis of DEGs

The expression levels of both lncRNAs and coding genes in each sample were calculated in FPKM values using RSEM (Li and Dewey, 2011). The formula used to calculate FPKM was as follows:

$$\begin{array}{l} FPKM=\frac{10^{6}C}{NL/10^{3}} \end{array}$$

We defined the expression level of gene A as FPKM(A). In the equation, C is the number of unique fragments aligned to gene A, N is the total number of unique fragments aligned to the reference genome, and L is the number of bases in the coding region of gene A. The FPKM method can eliminate the influences of differences in gene length and sequencing amounts on the calculation of gene expression. The calculated gene expression can be used to directly compare the expression of genes between different samples. The PoissonDis method (Audic and Claverie, 1997) was used to analyze the DEGs and DELs in the Qiufeng vs. Baiyu, Qiufeng vs. Nd-s^D^ and Qiufeng vs. Nd comparisons with filter parameters of a fold change ≥ 2.00 and a false discovery rate (*FDR*) ≤ 0.001.

### Analysis of LncRNA-miRNA Interactions

Given that lncRNAs may function as precursors of miRNAs, the lncRNA sequences were aligned to miRNAs published in miRBase (Griffiths-Jones et al., 2006) (http://www.mirbase.org/) in order to identify the known miRNA precursors and their secondary structures using BLASTN with a hit coverage threshold ≥90%. In addition, the interactions between lncRNAs and miRNAs were predicted using the Support Vector Machine (SVM)-based software miRanda (Wu et al., 2011).

### LncRNA Functions in Cis-/Trans-Regulation

The functions of lncRNAs can be reflected through analysis of the genes that are cis- and trans-regulated by the lncRNAs. Cis-regulation refers to the interaction of lncRNAs with neighboring target genes located either upstream or downstream. In this study, the 10 kb upstream and 20 kb downstream of the lncRNAs were investigated for potential coding genes. LncRNAs may play a trans-acting roles by binding to the sense strands of mRNA molecules. To further reveal the potential antisense lncRNA-mRNA interactions, we searched all antisense lncRNA-mRNA duplexes with complementary base pairing using RNAplex (Carrieri et al., 2012) with the ViennaRNA package, which predicts the best base-base pairing by using a minimum free energy algorithm according to thermodynamic features.

### Gene Ontology (GO) and Kyoto Encyclopedia of Genes and Genomes (KEGG) Pathway Analyses

Using Blast2GO software, GO analysis of the DEGs and target genes of the DELs was performed based on Nr annotation information deposited in NCBI (https://www.ncbi.nlm.nih.gov/). For pathway enrichment analysis, a hypergeometric test was used to identify pathways that were significantly enriched for the DEGs compared to the entire genome according to the KEGG (http://www.genome.jp/kegg/). To evaluate whether the candidate genes were enriched for a certain GO/KEGG pathway, we used a hypergeometric model to calculate the degrees of enrichment of the candidate genes; a smaller *P*-value was associated with greater enrichment of a gene in the corresponding pathway. Then, we performed FDR correction of the *P-*values, and a corrected *P* value <= 0.01 was set as the threshold for significant enrichment. Each *P*-value was calculated as follows:

$$\begin{array}{l} P=1-\overset{m-1}{\underset{i=0}{\sum }}\frac{\left(\begin{array}{c} M\\ i \end{array}\right)\left(\begin{array}{c} N-M\\ n-i \end{array}\right)}{\left(\begin{array}{c} N\\ n \end{array}\right)} \end{array}$$

## Data Availability Statement

The raw sequence reads has been uploaded to Sequence Read Archive (SRA) (http://www.ncbi.nlm.nih.gov/) with accession PRJNA634502.

## Author Contributions

BZ designed, supervised, and coordinated the study. JR, MW, XY, SZ, JL, LY, and ZY performed the study, analyzed, and interpreted the data. BZ and JR were involved in analyzing and processing the sequencing data. JR wrote the manuscript with the help of BZ. All authors read and approved the final manuscript.

## Conflict of Interest

The authors declare that the research was conducted in the absence of any commercial or financial relationships that could be construed as a potential conflict of interest.

## Supplementary Material

Supplementary Figure 1 Comparative analysis of lncRNAs and mRNAs in the PSG in *Bombyx mori*. Exon numbers of lncRNAs and mRNAs **(A)**. Transcript numbers of lncRNAs and mRNAs **(B)**. RNA lengths of lncRNAs and mRNAs **(C)**. The expression level of lncRNAs and mRNAs based on log10(FPKM) in Qiufeng **(D)**, Baiyu **(E)**, Nd-s^D^ **(F)** and Nd **(G)**.

Supplementary Figure 2 GO enrichment analysis of DEGs in Qiufeng vs. Baiyu **(A)**, Qiufeng vs. Nd-s^D^ **(B)** and Qiufeng vs. Nd **(C)**. The red bar represents the up-regulated target genes in accordance GO terms, the blue bar represents the down-regulated target genes in accordance GO terms.

Supplementary Figure 3 GO enrichment analysis for target genes of DELs in Qiufeng vs. Baiyu **(A)**, Qiufeng vs. Nd-s^D^ **(B)** and Qiufeng vs. Nd **(C)**. The red bar represents the up-regulated target genes in accordance GO terms, the blue bar represents the down-regulated target genes in accordance GO terms.

Supplementary Figure 4 Pathway enrichment analysis of differential expressed lncRNA. The enrichment results of protein processing in endoplasmic reticulum in Qiufeng vs. Baiyu **(A)**, Qiufeng vs. Nd-s^D^ **(B)** and Qiufeng vs. Nd **(C)**; the enrichment results of proteasome in Qiufeng vs. Baiyu **(D)**, Qiufeng vs. Nd-s^D^ **(E)** and Qiufeng vs. Nd **(F)**.

Supplementary Table 1 Total novel transcripts were assembled from the four libraries with StringTie. Transcripts and genes were labeled as TCONS and XLOC, respectively.

Supplementary Table 2 Identified lncRNAs can be compared to known transcripts. LncRNA transcripts identified in this study were labeled as LTCONS.

Supplementary Table 3 The expression level of lncRNA transcripts expressed only in Nd or Nd-s^D^.

Supplementary Table 4 Differential expression of mRNA genes in Qiufeng vs. Baiyu. Novel mRNA genes were labeled as MXLOC.

Supplementary Table 5 Differential expression of lncRNA genes in Qiufeng vs. Baiyu. LncRNA genes were labeled as LXLOC.

Supplementary Table 6 Differential expression of mRNAs in Qiufeng vs. Nd-s^D^.

Supplementary Table 7 Differential expression of lncRNAs in Qiufeng vs. Nd-s^D^.

Supplementary Table 8 Differential expression of mRNAs in Qiufeng vs. Nd.

Supplementary Table 9 Differential expression of lncRNAs in Qiufeng vs. Nd.

Supplementary Table 10 DEGs associated with protein proteolysis and programmed cell death.

Supplementary Table 11 Prediction results of lncRNAs may function as miRNA precursors.

Supplementary Table 12 The potential target genes of lncRNAs acting in cis/trans.

Supplementary Table 13 KEGG Pathway Enrichment Analysis of DEGs in Qiufeng vs. Baiyu, Qiufeng vs. Nd-s^D^ and Qiufeng vs. Nd.

Supplementary Table 14 KEGG Pathway Enrichment Analysis of DELs in Qiufeng vs. Baiyu, Qiufeng vs. Nd-s^D^ and Qiufeng vs. Nd.

Supplementary Table 15 The concentration and quality information of RNA samples.

## References

[B1] Amm I. Sommer T. Wolf D. H. (2014). Protein quality control and elimination of protein waste: the role of the ubiquitin-proteasome system. Biochim. Biophys. Acta 1843, 182–196. 10.1016/j.bbamcr.2013.06.031 23850760

[B2] Annilo T. Kepp K. Laan M. (2009). Natural antisense transcript of natriuretic peptide precursor A (NPPA): structural organization and modulation of NPPA expression. BMC Mol. Biol. 10:81. 10.1186/1471-2199-10-81 19671135 PMC2731763

[B3] Audic S. Claverie J. M. (1997). The significance of digital gene expression profiles. Genome Res. 7, 986–995. 10.1101/gr.7.10.986 9331369

[B4] Balakirev E. S. Ayala F. J. (2003). Pseudogenes: are they “junk” or functional DNA? Annu. Rev. Genet. 37, 123–151. 10.1146/annurev.genet.37.040103.103949 14616058

[B5] Beniaminov A. Westhof E. Krol A. (2008). Distinctive structures between chimpanzee and human in a brain noncoding RNA. RNA 14, 1270–1275. 10.1261/rna.1054608 18511501 PMC2441984

[B6] Bu D. Yu K. Sun S. Xie C. Geir S. Miao R.. (2011). NONCODE v3.0: integrative annotation of long noncoding RNAs. Nucleic Acids Res. 40, D210–D215. 10.1093/nar/gkr1175 22135294 PMC3245065

[B7] Buchberger A. Bukau B. Sommer T. (2010). Protein quality control in the cytosol and the endoplasmic reticulum: brothers in arms. Mol. Cell 40, 238–252. 10.1016/j.molcel.2010.10.001 20965419

[B8] Carninci P. Kasukawa T. Katayama S. Gough J. Frith M. C. Maeda N.. (2005). The transcriptional landscape of the mammalian genome. Science 309, 1559–1563. 10.1126/science.1112014 16141072

[B9] Carrieri C. Cimatti L. Biagioli M. Beugnet A. Zucchelli S. Fedele S.. (2012). Long non-coding antisense RNA controls Uchl1 translation through an embedded SINEB2 repeat. Nature 491, 454–457. 10.1038/nature11508 23064229

[B10] Chen R. T. Xiao Y. Liu Z. Li L. L. Lu Y. Jiao P.. (2019). Three vital RNA functions and interactions in the process of silk gland apoptosis in silkworm *Bombyx mori*. Arch. Insect Biochem. Physiol. 100:e21511. 10.1002/arch.21511 30417456

[B11] Chen Y. Dai H. Chen S. Zhang L. Long M. (2011). Highly tissue specific expression of Sphinx supports its male courtship related role in *Drosophila melanogaster*. PLoS ONE 6:e18853. 10.1371/journal.pone.0018853 21541324 PMC3082539

[B12] Coey C. T. Drohat A. C. (2017). Kinetic methods for studying DNA glycosylases functioning in base excision repair. Meth. Enzymol. 592:357. 10.1016/bs.mie.2017.03.016 28668127 PMC5761649

[B13] Derrien T. Johnson R. Bussotti G. Tanzer A. Djebali S. Tilgner H.. (2012). The GENCODE v7 catalog of human long noncoding RNAs: analysis of their gene structure, evolution, and expression. Genome Res. 22, 1775–1789. 10.1101/gr.132159.111 22955988 PMC3431493

[B14] Finn R. D. Penelope C. Eberhardt R. Y. Eddy S. R. Jaina M. Mitchell A. L.. (2015). The Pfam protein families database: towards a more sustainable future. Nucleic Acids Res. 44, D279–D285. 10.1093/nar/gkv1344 26673716 PMC4702930

[B15] Gong C. Maquat L. E. (2011). lncRNAs transactivate STAU1-mediated mRNA decay by duplexing with 3′ UTRs via Alu elements. Nature 470, 284–288. 10.1038/nature09701 21307942 PMC3073508

[B16] Griffiths-Jones S. Grocock R. J. Van Dongen S. Bateman A. Enright A. J. (2006). miRBase: microRNA sequences, targets and gene nomenclature. Nucleic Acids Res 34, D140–D144. 10.1093/nar/gkj112 16381832 PMC1347474

[B17] Hu W. Chen Y. Lin Y. Xia Q. (2019a). Developmental and transcriptomic features characterize defects of silk gland growth and silk production in silkworm naked pupa mutant. Insect Biochem. Mol. Biol. 111:103175. 10.1016/j.ibmb.2019.05.010 31150761

[B18] Hu W. Lu W. Wei L. Zhang Y. Xia Q. (2019b). Molecular nature of dominant naked pupa mutation reveals novel insights into silk production in *Bombyx mori*. Insect Biochem. Mol. Biol. 109, 52–62. 10.1016/j.ibmb.2019.04.006 30954682

[B19] Igor M. Aroul R. Ana S. B. Natalie C. Alexandre A. J. N. (2007). Repression of the human dihydrofolate reductase gene by a non-coding interfering transcript. Nature 445, 666–670. 10.1038/nature05519 17237763

[B20] Ilik I. A. Maticzka D. Georgiev P. Gutierrez N. M. Backofen R. Akhtar A. (2017). A mutually exclusive stem-loop arrangement in roX2 RNA is essential for X-chromosome regulation in Drosophila. Genes Dev. 31, 1973–1987. 10.1101/gad.304600.117 29066499 PMC5710142

[B21] Inoue S. Kanda T. Imamura M. Quan G. X. Kojima K. Tanaka H.. (2005). A fibroin secretion-deficient silkworm mutant, Nd-sD, provides an efficient system for producing recombinant proteins. Insect Biochem. Mol. Biol. 35, 51–59. 10.1016/j.ibmb.2004.10.002 15607655

[B22] Jing Z. Ohsumi T. K. Kung J. T. Ogawa Y. Grau D. J. Sarma K.. (2010). Genome-wide identification of polycomb-associated RNAs by RIP-seq. Mol. Cell. 40, 939–953. 10.1016/j.molcel.2010.12.011 21172659 PMC3021903

[B23] Kim D. Langmead B. Salzberg S. L. (2015). HISAT: a fast spliced aligner with low memory requirements. Nat. Methods 12, 357–360. 10.1038/nmeth.3317 25751142 PMC4655817

[B24] Kimoto M. Tsubota T. Uchino K. Sezutsu H. Takiya S. (2014). LIM-homeodomain transcription factor Awh is a key component activating all three fibroin genes, fibH, fibL and fhx, in the silk gland of the silkworm, *Bombyx mori*. Insect Biochem. Mol. Biol. 56, 29–35. 10.1016/j.ibmb.2014.11.003 25449130

[B25] Kudo T. Katayama T. Imaizumi K. Yasuda Y. Takeda M. (2002). The unfolded protein response is involved in the pathology of alzheimer's disease. Ann. N. Y. Acad. Sci. 977, 349–355. 10.1111/j.1749-6632.2002.tb04837.x 12480772

[B26] Lei K. Yong Z. Ye Z. Q. Liu X. Q. Zhao S. Q. Wei L.. (2007). CPC: assess the protein-coding potential of transcripts using sequence features and support vector machine. Nucleic Acids Res. 35, W345–W349. 10.1093/nar/gkm391 17631615 PMC1933232

[B27] Li B. Dewey C. N. (2011). RSEM: accurate transcript quantification from RNA-Seq data with or without a reference genome. BMC Bioinformatics 12, 323 10.1186/1471-2105-12-323 21816040 PMC3163565

[B28] Li D. Wang Y. Zhang K. Jiao Z. Zhu X. Skogerboe G.. (2011). Experimental RNomics and genomic comparative analysis reveal a large group of species-specific small non-message RNAs in the silkworm *Bombyx mori*. Nucleic Acids Res. 39, 3792–3805. 10.1093/nar/gkq1317 21227919 PMC3089462

[B29] Li D. D. Liu Z. C. Huang L. Jiang Q. L. Zhang K. Qiao H. L.. (2014). The expression analysis of silk gland-enriched intermediate-size non-coding RNAs in silkworm *Bombyx mori*. Insect. Sci. 21, 429–438. 10.1111/1744-7917.12063 24124013

[B30] Li G.-M. (2008). Mechanisms and functions of DNA mismatch repair. Cell Res. 18, 85–98. 10.1038/cr.2007.115 18157157

[B31] Li J. Y. Cai F. Ye X. G. Liang J. S. Li J. K. Wu M. Y.. (2017). Comparative proteomic analysis of posterior silk glands of wild and domesticated silkworms reveals functional evolution during domestication. J. Proteome Res. 16, 2495–2507. 10.1021/acs.jproteome.7b00077 28569067

[B32] Li R. Li Y. Kristiansen K. Wang J. (2008). SOAP: short oligonucleotide alignment program. Bioinformatics 24, 713–714. 10.1093/bioinformatics/btn025 18227114

[B33] Liang S. Luo H. Bu D. Zhao G. Yu K. Zhang C.. (2013). Utilizing sequence intrinsic composition to classify protein-coding and long non-coding transcripts. Nucleic Acids Res. 41:e166. 10.1093/nar/gkt646 23892401 PMC3783192

[B34] Matus S. Glimcher L. H. Hetz C. (2011). Protein folding stress in neurodegenerative diseases: a glimpse into the ER. Curr. Opin. Cell. Biol. 23, 239–252. 10.1016/j.ceb.2011.01.003 21288706

[B35] Mercer T. R. Dinger M. E. Mattick J. S. (2009). Long non-coding RNAs: insights into functions. Nat. Rev. Genetics 10, 155–159. 10.1038/nrg2521 19188922

[B36] Miao Z. Wang S. Zhang J. Wei P. Guo L. Liu D.. (2018). Identification and comparison of long non-conding RNA in jinhua and landrace pigs. Biochem. Biophys. Res. Commun. 506, 765–771. 10.1016/j.bbrc.2018.06.028 29890140

[B37] Michalak M. (2010). Quality control in the endoplasmic reticulum. Semin. Cell Dev. Biol. 21:471. 10.1016/j.semcdb.2010.03.005 20304087

[B38] Mori K. Tanaka K. Kikuchi Y. Waga M. Waga S. Mizuno S. (1995). Production of a chimeric fibroin light-chain polypeptide in a fibroin secretion-deficient naked pupa mutant of the silkworm *Bombyx mori*. J. Mol. Biol. 251, 217–228. 10.1006/jmbi.1995.0429 7643398

[B39] Mulvey B. B. Olcese U. Cabrera J. R. Horabin J. I. (2014). An interactive network of long non-coding RNAs facilitates the drosophila sex determination decision. Biochim Biophys Acta 1839, 773–784. 10.1016/j.bbagrm.2014.06.007 24954180 PMC4134978

[B40] Ohno K. Sawada J.-I. Takiya S. Mai K. Matsumoto A. Tsubota T.. (2013). Silk gland factor-2, involved in fibroin gene transcription, consists of LIM homeodomain, LIM-interacting, and single-stranded DNA-binding proteins. J. Biol. Chem. 288, 31581–31591. 10.1074/jbc.M113.514471 24022586 PMC3814754

[B41] Okazaki Y. Furuno M. Kasukawa T. Adachi J. Bono H. Kondo S.. (2002). Analysis of the mouse transcriptome based on functional annotation of 60,770 full-length cDNAs. Nature 420, 563–573. 10.1038/nature01266 12466851

[B42] Pandey P. Wang M. Baldwin I. T. Pandey S. P. Groten K. J. B. G. (2018). Complex regulation of microRNAs in roots of competitively-grown isogenic Nicotiana attenuata plants with different capacities to interact with arbuscular mycorrhizal fungi. BMC Genomics 19:937. 10.1186/s12864-018-5338-x 30558527 PMC6296096

[B43] Parkhomchuk D. Borodina T. Amstislavskiy V. Banaru M. Hallen L. Krobitsch S.. (2009). Transcriptome analysis by strand-specific sequencing of complementary DNA. Nucleic Acids Res. 37:e123. 10.1093/nar/gkp596 19620212 PMC2764448

[B44] Pertea M. Pertea G. M. Antonescu C. M. Chang T.-C. Mendell J. T. Salzberg S. L. (2015). StringTie enables improved reconstruction of a transcriptome from RNA-seq reads. Nat. Biotechnol. 33, 290–295. 10.1038/nbt.3122 25690850 PMC4643835

[B45] Samata M. Akhtar A. (2018). Dosage compensation of the X chromosome: A complex epigenetic assignment involving chromatin regulators and long noncoding RNAs. Annu. Rev. Biochem. 87, 323–350. 10.1146/annurev-biochem-062917-011816 29668306

[B46] Sancar A. Reardon J. T. (2004). Nucleotide excision repair in *E. coli* and man. Adv. Protein Chem. 69, 43–71. 10.1016/S0065-3233(04)69002-4 15588839

[B47] Takei F. Oyama F. Kimura K. Hyodo A. Mizuno S. Shimura K. (1984). Reduced level of secretion and absence of subunit combination for the fibroin synthesized by a mutant silkworm, Nd(2). J. Cell. Biol. 99, 2005–2010. 10.1083/jcb.99.6.2005 6209286 PMC2113558

[B48] Tiash S. Chowdhury E. H. (2018). siRNAs targeting multidrug transporter genes sensitise breast tumour to doxorubicin in a syngeneic mouse model. J. Drug Target. 27, 325–337. 10.1080/1061186X.2018.1525388 30221549

[B49] Tsai K. Courtney D. G. Kennedy E. M. Cullen B. R. (2018). Influenza A virus-derived siRNAs increase in the absence of NS1 yet fail to inhibit virus replication. RNA 9:1044. 10.1261/rna.066332.118 29903832 PMC6097656

[B50] Wang S. Ge W. Luo Z. Guo Y. Jiao B. Qu L.. (2017). Integrated analysis of coding genes and non-coding RNAs during hair follicle cycle of cashmere goat (Capra hircus). BMC Genomics 18:767. 10.1186/s12864-017-4145-0 29020916 PMC5637055

[B51] Wang S. You Z. Feng M. Che J. Zhang Y. Qian Q.. (2016). Analyses of the molecular mechanisms associated with silk production in silkworm by iTRAQ-based proteomics and RNA-sequencing-based transcriptomics. J. Proteome Res. 15, 15–28. 10.1021/acs.jproteome.5b00821 26626507

[B52] Wang S. H. You Z. Y. Ye L. P. Che J. Qian Q. Nanjo Y.. (2014). Quantitative proteomic and transcriptomic analyses of molecular mechanisms associated with low silk production in silkworm *Bombyx mori*. J. Proteome Res. 13, 735–751. 10.1021/pr4008333 24428189

[B53] Wilusz J. E. Sunwoo H. Spector D. L. (2009). Long noncoding RNAs: functional surprises from the RNA world. Genes Dev. 23, 1494–1504. 10.1101/gad.1800909 19571179 PMC3152381

[B54] Wu Y. Cheng T. Liu C. Liu D. Zhang Q. Long R.. (2016). Systematic identification and characterization of long non-coding rnas in the silkworm, *Bombyx mori*. PLoS ONE 11:e0147147. 10.1371/journal.pone.0147147 26771876 PMC4714849

[B55] Wu Y. Wei B. Liu H. Li T. Rayner S. (2011). MiRPara: a SVM-based software tool for prediction of most probable microRNA coding regions in genome scale sequences. BMC Bioinf. 12:107 10.1186/1471-2105-12-107 PMC3110143 21504621

[B56] Yazgan O. Krebs J. E. (2007). Noncoding but nonexpendable: transcriptional regulation by large noncoding RNA in eukaryotes. Biochem. Cell Biol. 85, 484–496. 10.1139/O07-061 17713583

[B57] Yuya O. Sun B. K. Lee J. T. (2008). Intersection of the RNA interference and X-inactivation pathways. Science 320, 1336–1341. 10.1126/science.1157676 18535243 PMC2584363

[B58] Zhao J. Sun B. K. Erwin J. A. Song J.-J. Lee J. T. (2008). Polycomb proteins targeted by a short repeat RNA to the mouse X chromosome. Science 322, 750–756. 10.1126/science.1163045 18974356 PMC2748911

[B59] Zhou Q.-Z. Fang S.-M. Zhang Q. Yu Q.-Y. Zhang Z. (2018). Identification and comparison of long non-coding RNAs in the silk gland between domestic and wild silkworms. Insect Sci. 25, 604–616. 10.1111/1744-7917.12443 28111905

[B60] Zhu Xs S. Y. Xu S. Q. (2009). Application of *Bombyx mori* as model organism in modern biology. Lab. Anim. Comp. Med. 29, 61–65. 10.3969/j.issn.1004-8448.2009.01.017

