# Supplementary figures and images for: Comparative mRNA and LncRNA Analysis of the Molecular Mechanisms Associated With Low Silk Production in Bombyx mori
Source: Front Genet. 2021 Jan 21;11:592128. doi: 10.3389/fgene.2020.592128 (PMC7859555; doi:10.3389/fgene.2020.592128)

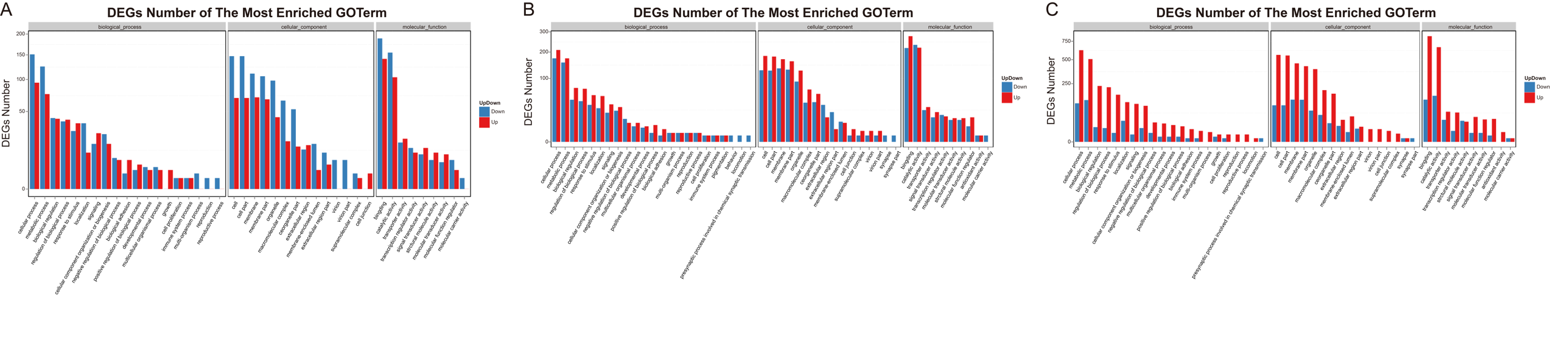

Supplement: Supplementary Figure 2 — GO enrichment analysis of DEGs in Qiufeng vs. Baiyu (A), Qiufeng vs. Nd-sD (B) and Qiufeng vs. Nd (C). The red bar represents the up-regulated target genes in accordance GO terms, the blue bar represents the down-regulated target genes in accordance GO terms. [file Image_2.TIF]

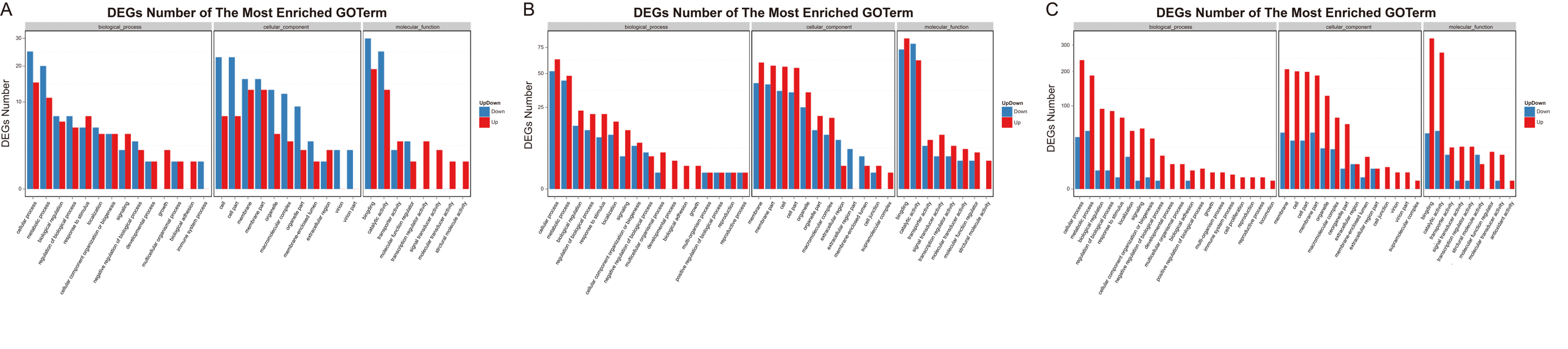

Supplement: Supplementary Figure 3 — GO enrichment analysis for target genes of DELs in Qiufeng vs. Baiyu (A), Qiufeng vs. Nd-sD (B) and Qiufeng vs. Nd (C). The red bar represents the up-regulated target genes in accordance GO terms, the blue bar represents the down-regulated target genes in accordance GO terms. [file Image_3.TIF]
